# Supplementary material for: HPV induced R-loop formation represses innate immune gene expression while activating DNA damage repair pathways
Source: PLoS Pathog. 2024 Aug 23;20(8):e1012454. doi: 10.1371/journal.ppat.1012454 (PMC11376575; doi:10.1371/journal.ppat.1012454)
Supplement: Attachment — Submitted filename: Letter to Editors Revision 7-2.docx [file ppat.1012454.s009.docx]

PloS Pathogens

Dear Editors

We are returning to you a revised version of our manuscript (PPATHOGENS-D-24-01051), “HPV induced R-loop formation represses innate immune gene expression while activating DNA damage repair pathways,” for consideration for publication. The two reviewers thought the manuscript was interesting, and we have revised it to address their points. We have also included two additional supplemental figures to address question # 3 by reviewer #2 about our quality control methods to assure the differences are biologically significant.

Reviewer #1

1). Consider that RNase H1 impacts viral maintenance and transcription as part of the interpretation of results:

Our previous studies have shown that R-loops are formed at the early promoter as well as termination sequences on viral genomes. Overexpression of RNAse H1 reduces R-loops and impairs viral transcription by 50 to 60%, along with ~50% lower levels of viral episomes (Templeton and Laimins PNAS 2023). Since the overexpression of RNase H1 reduces the expression of early viral proteins, and E6 is responsible for the increased levels of R-loop, decreasing its expression would act to reduce R-loop levels further. Furthermore, studies from a number of groups have implicated R-loops in regulating gene expression, such as polycomb-regulated genes where overexpression of RNAse H1 led to decreased levels of R-loops and resulted in impaired expression of some genes but higher levels of others (Skourtit-Stathaki et al Mo. Cell. 2019 and reviewed in Cerritelli et al. 2022) and this is now included in the Discussion page 21, lines 519 – 526. This R-loop directed regulation of expression occurs in conjunction with the action of cellular proteins, and we believe this occurs in our studies as well. We believe that R-loops must act together with other factors, including modified chromatin, to regulate the expression of genes in specific pathways, such as the innate immune response, and it is possible an early viral protein could be one of these regulators. Our studies show a tight association between the presence of R-loops and the regulation of expression of specific pathways so that any contribution of a viral or cellular factor would be linked. We have now included a detailed discussion of a possible role for viral proteins in acting together with R-loops in regulating expression on page 11-12, lines 275 – 278 in the Results and on page 18, lines 446 – 450; page 21, lines 527 - 529 in the Discussion.

2). Peak-calling algorithms to calculate the overlap of R-loops and modified histones on chromatin

We have now added a statement explaining our methodologies in the results section (lines 318 - 322): “We performed peak calling algorithms on each of the modified histones pulldown experiments using the MACS peak calling algorithm. We controlled for off-target pulldowns by background subtracting respective input control samples isolated from each of the cell lines (HFK and CIN 612 cells). The called peaks were then assigned a relative genomic location using the HOMER motif recognition software”. Once genomic locations were assigned, only those associated within 2kb of a gene’s coding region were reported, thereby removing peaks associated with distal intergenic regions. The remaining genes with reported peaks from each of the modified histone pulldowns were then compared to the genes associated with R-loop peaks identified in the same manner. This comparison is what makes up the data for the Venn Diagrams in Figures 5 and 6. A detailed discussion of the methodologies and analysis of the peak calling assays from each of the modified histone pulldowns is now included in the Results and Materials and Methods sections.

Reviewer#2

1). DRIP-seq data shows 3 major peaks at 1kb upstream of TSS, gene body, and 1 kb downstream of TES. This seems unusual, so compare it to other published DRIP-seq data sets.

The profile of our DRIP-seq reads is similar to that shown in Promonet et al. Nat. Comm. 2020. The peak found in our studies near the TSS is ~1kb upstream, similar to that seen in Promonet et al. 2020. In addition, our plotted X-axis is only 2kb upstream and downstream of the coding sequence, while other studies have ranged from 10kb to 50kb. We believe this significantly affects how the profile is visualized, making promoter associated R-loops appear close to start sites. Additionally, peaks within 2kb of a gene’s start or end site have commonly been characterized as peaks associated with the promoter or termination sequence (see comment #4). The two peaks at and near the TES are similar to what has been published in a number of other studies, while other studies have described R-loop read profiles that detect increased read levels upstream of the TSS (e.g., Sessa et al. Embo Press. 2021, Munden et al. Journal of Mol. Biol. 2022). We reference the similarity between our study and others in the Results section (lines 155 - 156)

2). A minority of genes had unique R-loop distributions. What are these genes?

As demonstrated by the XY correlation plots and heatmaps of Pearson coefficient values among the S9.6 pulldown replicates (both from HFK and CIN 612 cells; Supp Fig 7), there were high correlations where the S9.6 reads were found along the genome (Pearson coefficient between 0.97 and 0.99). The very small variation in these analyses led to our statement that only a minority of genes had unique R-loop distributions. Additionally, we found some genes that had associated R-loops but at a distance of up to 50kb from the coding sequence. We sought to exclude these from our studies but may investigate them in more detail in the future.

3). In DRIP-seq data, how were samples normalized? How do biological replicates correlate with each other?

Our DRIP-seq data was normalized using input control nucleic acids that underwent the same treatment as the nucleic acids used for our S9.6 pulldown assays. This input DNA was used to subtract the background and normalize our S9.6 pulldown samples for peak calling as shown in Figure 2 as well as for the individual depth graphs seen in Figure 1E. We have included additional Supplemental Figures 7 and 8 to show the correlations of our biological replicates and provided a description of this point on lines 652-657 and 691- 694 in the materials and methods section. These figures demonstrate Pearson coefficients between our independent sequencing replicates from our S9.6, H3K36me3, H3K9me3, and γH2AX pulldowns. We also performed principal component analyses on each of these subsets to ensure proper clustering of our replicates for each pulldown experiment. These data support the strong agreement between each of the biological replicates presented in this study.

4). Authors state 60% of DRIP-seq data are distributed in genic regions but in apparent disagreement with data presented (Figure 1D and Figure 2C).

Figure 1D represents averaged read counts from both biological replicates of the S9.6 pulldown. Figure 2C represents the genomic location assigned to each called peak based on the analysis using the MACS peak calling algorithm. The MACS peak calling algorithm has built-in cut-offs for enrichments over 50-fold and less than 5-fold over our input control data. These cut-offs could result in discrepancies between the heatmap in Figure 1D and the pie chart in Figure 2C. We mistakenly labeled Figure 2C as “R-loop read distribution” when it should read “R-loop peak distribution”. We apologize for the confusion this caused. Changes have been made to the Results section and Figure Legends (fig 2) to correct this mistake (lines 182, 184, and 188)

We have used the word “genic” to refer to R-loops formed near a gene’s coding region. This term has been used in other studies (Reviewed in Chedin, Trend Genet., 2016; Castillo-Guzman et al. DNA Repair, 2021), and we have updated the text on lines 156-157 to define this term more clearly. Additionally, a distance of 2kb has been used to associate peaks with a gene’s start site or termination site for a number of studies (Griffon et al, NAR, 2015; Shin et al, DNA Research, 2016; Hsu et al, NAR, 2024).

5). Are R-loops a consequence or cause of altered expression? Do genes in subsets have specific sequence features such as GC skew or G4 quadraplexes?

The reviewer raises the question as to whether R-loops are the consequence rather than the cause of the altered expression of genes at which they form. This is, in fact, the reason we analyzed cells through RNase H1 overexpression, resulting in reduced R-loop levels, and correlated that with RNA-seq data with and without RNAse H1. We believe this data shows a correlation of R-loops with either activation or repression of genes in specific pathways and is consistent with published studies (Skourti-Stathaki et al, Mol Cell, 2019).

With respect to GC skew or G4 quadruplexes, we have scanned the R-loop regions of innate immune genes and DNA damage repair loci. While the averaged sequences of all genes differentially regulated are not GC skewed, there are examples of genes both downregulated and upregulated with R-loops present on or near long stretches of GCs (CpG islands). In particular, innate immune genes tended to have robust enrichments of R-loop formation near CpG islands in the CIN 612 cells. Some examples of these genes included IL6R, DDX58, and IRF4.

In addition, G4 quadruplexes have been reported to influence R-loop stability, and we have included a description of this in the Discussion on page 21, lines 506 - 513. Definitive identification of G4 quadruplexes in R-loop regions will, however, require the use of an antibody specific to these structures in ChIP assays. The possibility that other non-B DNA structures might cooperate with R-loops to facilitate differential gene expression is an important area of future research. We now include a statement regarding GC skew and G4 quadraplexes in the Discussion on pg. 21.

6). Surprising result that H3K9me3 in HFKs is associated with increased mRNA levels.

We have performed a binary analysis of the RNA-seq data with the H3K9me3 negative and positive genes (FKPM > 0) in the HFKs using ChIP-seq, and found about a 0.33 fold increase in average read counts from 3,000 to 4,000 associated with increased expression. In comparison to the other histone modifications and R-loops associated gene expression, we found increases ranging from 2 to 10-fold. We believe the difference may be statistically significant for H3K9me3 but are unsure of the biological significance. Mention of this result was added to the Results, page 14, lines 335-337. In addition, H3K9me3 has been previously linked with increased transcription on certain alleles in different cell lines (Squazzo et al. Genome Res. 2006), which may be relevant to our analyses.

**Minor issues:**

Reviewer #1

1) Provide details on how Venn diagrams overlaps calculated? Genome size?

Venn diagram overlap representation factors (RF) were calculated by using the mapped RNA read counts for each cell line. All non-coding (lnRNAs, miRNAs, etc) and coding genes were added together for total genome size. Furthermore, we standardized our genome size between both samples by using the estimated number of coding and non-coding genes from the CHESS database (63,755; [http://ccb.jhu.edu/chess](http://ccb.jhu.edu/chess)), and this is described in the Materials and Methods section, page 29, lines 713-714. RFs of 1 or greater represent more overlap than expected from random distributions, while RFs of less than 1 represent less overlap than expected. We have now labeled the RF and p-value associated with the Venn Diagrams in Figures 5B and 6B.

2) Venn diagrams rely on arbitrary cut-offs ..describe

Please see reviewer #1's comment #2 on how we determined R-loop-associated genes that either contained the modified histone of interest or did not.

3) Reviewer #1 asks us to temper our conclusions about the association of R-loops, chromatin, and gene expression.

We have modified our statements in the Discussion (lines 536-539) and lines 42 and 44 in the Abstract about the association of R-loops, chromatin modifications, and expression to say that our studies may indicate a potential linkage, but that further studies are needed.

We hope these changes address the reviewers' concerns, but we are happy to make additional changes as requested. We are also happy to publish the peer review history.

Sincerely
